# Convolutional neural network for colorimetric glucose detection using a smartphone and novel multilayer polyvinyl film microfluidic device

**DOI:** 10.1038/s41598-024-79581-y

**Published:** 2024-11-17

**Authors:** Mithun Kanchan, Prasad Kisan Tambe, Sanjay Bharati, Omkar S Powar

**Affiliations:** 1 Department of Mechanical and Industrial Engineering, Manipal Institute of Technology, Manipal Academy of Higher Education, Manipal, 576104 Karnataka India; 2 Department of Nuclear Medicine, Manipal College of Health Professions, Manipal Academy of Higher Education, Manipal, 576104 Karnataka India; 3 Department of Biomedical Engineering, Manipal Institute of Technology, Manipal Academy of Higher Education, Manipal, 576104 Karnataka India

**Keywords:** Colorimetry, Convolution neural networks, Glucose, Image processing, Deep learning, Microfluidics, Smartphone, Fluidics, Biomedical engineering, Biochemical reaction networks, Data processing, Machine learning

## Abstract

Detecting glucose levels is crucial for diabetes patients as it enables timely and effective management, preventing complications and promoting overall health. In this endeavor, we have designed a novel, affordable point-of-care diagnostic device utilizing microfluidic principles, a smartphone camera, and established laboratory colorimetric methods for accurate glucose estimation. Our proposed microfluidic device comprises layers of adhesive poly-vinyl films stacked on a poly methyl methacrylate (PMMA) base sheet, with micro-channel contours precision-cut using a cutting printer. Employing the gold standard glucose-oxidase/peroxidase reaction on this microfluidic platform, we achieve enzymatic glucose determination. The resulting colored complex, formed by phenol and 4-aminoantipyrine in the presence of hydrogen peroxide generated during glucose oxidation, is captured at various glucose concentrations using a smartphone camera. Raw images are processed and utilized as input data for a 2-D convolutional neural network (CNN) deep learning classifier, demonstrating an impressive 95% overall accuracy against new images. The glucose predictions done by CNN are compared with ISO 15197:2013/2015 gold standard norms. Furthermore, the classifier exhibits outstanding precision, recall, and F1 score of 94%, 93%, and 93%, respectively, as validated through our study, showcasing its exceptional predictive capability. Next, a user-friendly smartphone application named “GLUCOLENS AI” was developed to capture images, perform image processing, and communicate with cloud server containing the CNN classifier. The developed CNN model can be successfully used as a pre-trained model for future glucose concentration predictions.

## Introduction

Diabetes mellitus, a metabolic disorder characterized by elevated blood glucose levels, is a major health concern, contributing to over 1.5 million deaths globally and acting as a significant factor in cardiovascular diseases like heart attacks and strokes^1^. Monitoring blood glucose is crucial for managing diabetes, especially since glucose levels fluctuate due to various factors, such as insulin administration or fasting^2,3^. A significant drawback of traditional home glucometers is their reliance on electrochemical principles, which can lead to discrepancies between blood and plasma glucose levels. Factors such as hematocrit variations, oxygen levels, and dehydration can result in inaccurate readings. While glucometers are useful for home monitoring, they exhibit deviations from the gold standard, with errors as high as 30–40%, far exceeding the acceptable 5% threshold. This highlights the need for improved methods that provide more reliable glucose estimations.

Colorimetric detection has gained prominence as an alternative approach for glucose measurement, where glucose concentration correlates with color intensity. Traditional methods involve separating plasma from blood, followed by an enzymatic reaction between glucose and glucose oxidase, producing gluconic acid and hydrogen peroxide. The hydrogen peroxide reacts with peroxidase and a chromogenic indicator, forming a colored product^4,5^. The intensity of the color is measured spectrophotometrically, providing an accurate glucose concentration estimate^6,7^.

Recent advances in healthcare have introduced Point-of-Care (POC)^8^ devices employing microfluidic technologies, which offer rapid, efficient, and cost-effective platforms for diagnosis, especially in resource-limited settings^9,10^. These devices minimize sample and reagent requirements while enhancing sensitivity. Specifically, the microfluidic device for enzymatic reactions should be a single-step fabrication process with direct indication, and the equipment used to measure color intensity must ensure accuracy and scalability. Paper-based microfluidic devices (µPADs) have gained attention for their low cost, simplicity, and disposability^11–14^. Despite these features, issues such as washing effects^15^, where reagents and enzymes migrate toward the edges of the testing zone, and non-homogeneous color formation due to enzymes or spotted reagents failing to attach securely to the paper’s surface, pose significant challenges^16^. Other limitations include porosity, overflowing, and inconsistent color development, all of which affect the reliability of the results. Strategies like immobilizing reagents, treating paper with functionalized nanoparticles, and oxidation have been explored to address these challenges^17–21^. Nevertheless, paper-based devices often require multi-step fabrication processes, such as wax printing and thermal treatment, making them labour-intensive and time-consuming. Plastic-based microfluidic platforms, using materials like PDMS and polyester, provide advantages over paper-based devices by achieving color homogeneity and improved surface treatment, with polyester film devices favored for their single-step fabrication and capillary-driven fluid movement, applicable in PCR and colorimetric assays for glucose and proteins^22–28^. In point-of-care (POC) applications, methods like standard addition assays, flatbed scanners, and smartphones have been explored for color intensity analysis^29^; however, while scanners ensure consistent imaging, their lack of portability limits use^30,31,32,33,34,35,36,37,38^. Smartphones offer a more practical solution but face challenges from variations in settings that can affect accuracy^39–43^. Although deep learning and machine learning models have been proposed to correct environmental variations, issues remain regarding robust dataset development and feature extraction^39,40,44,45^.

**Fig. 1:**
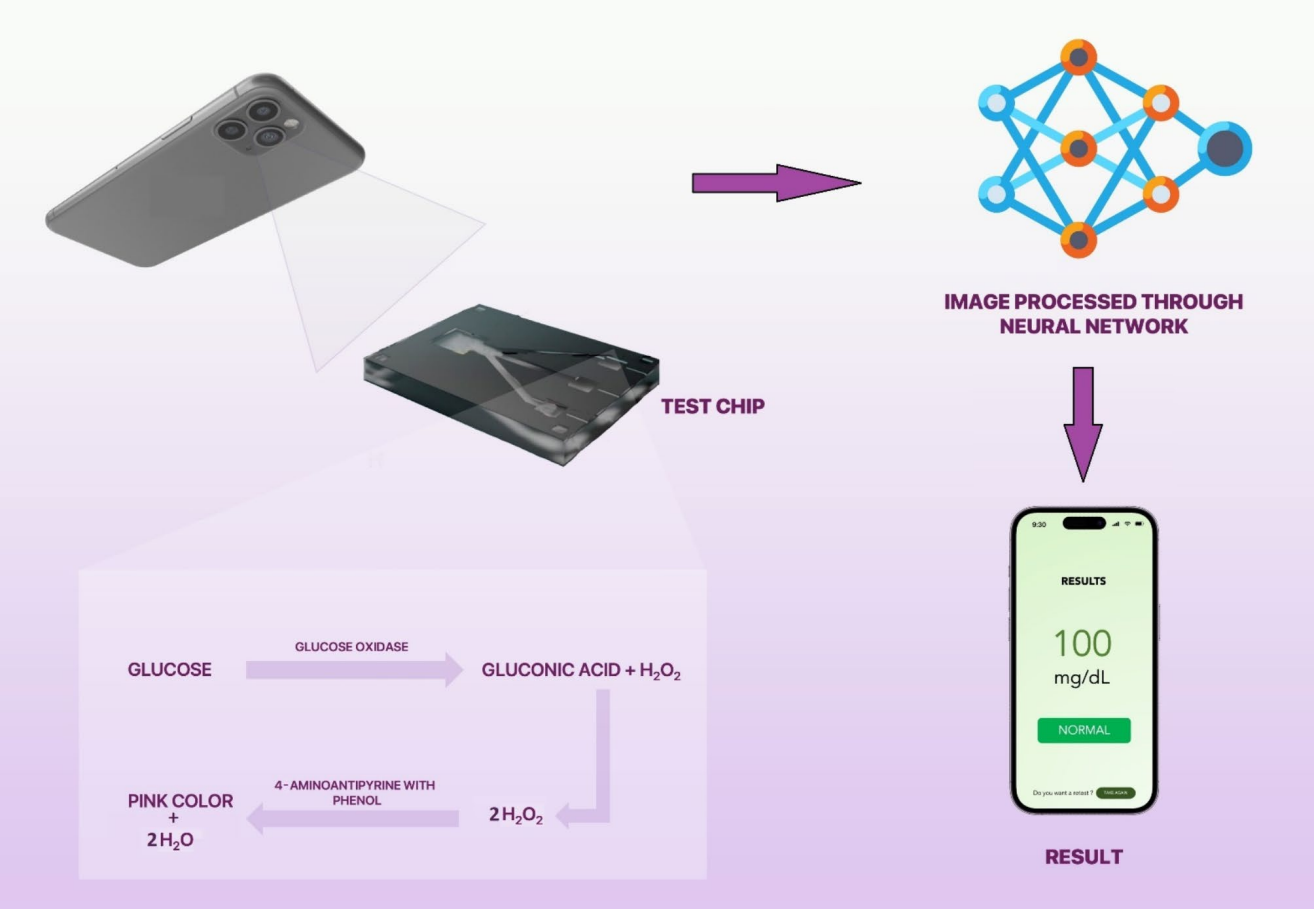
The overview of the present study. A PMMA base plate multi-layered adhesive-coated poly-vinyl film microfluidic device is shown in the middle. The Glucose Oxidase-Peroxidase reaction for colorimetric estimation of glucose is carried out in the device. The output color intensity for glucose samples is captured with the help of a smartphone containing the “GLUCOLENS AI” application. The processed images are classified using a Convolution-Neural Network (CNN) deep learning classifier and the resulting glucose concentration is displayed in the application.

Despite advancements in microfluidic devices and colorimetric detection technologies, a significant need remains for a mass-producible, rapid, low-cost, portable POC device that accurately measures glucose levels without requiring additional instruments or trained personnel. This research presents a novel approach to glucose estimation using an affordable point-of-care diagnostic device that employs microfluidic principles and the gold standard glucose-oxidase/peroxidase (GOD/POD) reaction, operates independently of external calibration, enhancing both accuracy and sensitivity. The device can handle up to three plasma glucose samples simultaneously, using capillary action to propel the enzyme reaction for glucose detection. Our method improves upon traditional paper-based lateral flow strips by utilizing a three-dimensional microfluidic channel for more reliable enzyme reactions. It also addresses common issues such as inadequate blood samples, strip malfunctions, and contamination risks. The study uses a smartphone camera for real-time image capture, achieving a remarkable 95% overall accuracy with a 2-D CNN, alongside strong performance metrics. Enhanced image processing is accomplished through advanced CNN architectures fine-tuned for small sample size datasets typically encountered in this type of research, resulting in improved accuracy in detecting subtle colour changes in images, especially under varying environmental conditions. Furthermore, the user-friendly smartphone application, “GLUCOLENS AI,” facilitates seamless interaction and data communication, making the solution accessible and practical for non-specialists. Leveraging a deep-learning model for colorimetric detection ensures accurate glucose monitoring in various settings without requiring trained personnel, laboratory equipment, or additional instrumentation. An overview of the present study is provided in Fig. 1.

## Experimental work

### Chemicals and assay standardization

In our research, the Mybio Glucose Test Kit from Mylab Discovery Solutions Pvt. Ltd., India, was employed. This kit comprised a Glucose enzyme reagent, consisting of Phosphate buffer, glucose oxidase, Peroxidase, 4-amino antipyrine, and Phenol, along with a Glucose standard. The fundamental operational principle of the test kit is based on the GOD/POD method (glucose oxidase-peroxidase coupled method) for glucose detection. To adhere to the established normal range of glucose levels in healthy individuals, specifically 70 to 110 mg/dL (fasting) and up to 130 mg/dL (post-prandial), a series of standard laboratory glucose solutions (D-galactose, SRL chemicals, India) were meticulously prepared. These solutions ranged from 50 mg/dL to 200 mg/dL, with an incremental concentration difference of 10 mg/dL, resulting in a total of 16 samples. To ensure precision and minimize experimental errors, all procedures were executed using calibrated micropipettes with volumes ranging from 1.0 µL to 10 µL and 100 µL to 1000 µL. Spectrophotometric analysis was employed to confirm the accuracy of the prepared glucose solutions (50 mg/dL to 200 mg/dL) with the specified test kit. Subsequently, following confirmation, we proceeded to assess the levels of these samples using our microfluidic device^46–48^.

### Fabrication of the microfluidic device

**Fig. 2:**
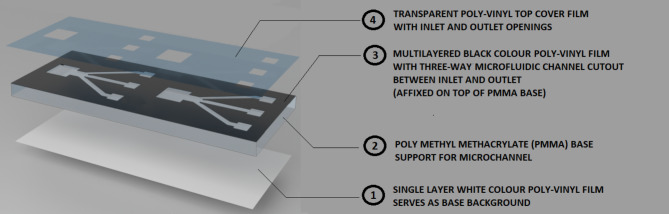
The fabrication steps involved in the development of adhesive-coated poly-vinyl films stacked on poly methyl methacrylate (PMMA) baseplate microfluidic device. Four layers that make up the microfluidic device are shown. (1) A single layer of white color poly-vinyl film affixed to the bottom of the PMMA base thus serving as background during image capture. (2) PMMA transparent baseplate having its top side affixed with poly-vinyl films. (3) Single or multi-layered (depending upon the required channel depth) adhesive-coated black vinyl films on which microfluidic channels are cut out using a cutting printer. (4) The adhesive-coated transparent vinyl film containing the openings for inlet and outlet wells thus sealing the microfluidic channel.

The microfluidic device used in this study is constructed from layers of adhesive-coated poly-vinyl (PVC) films stacked atop each other as shown in Fig. 2. The layout and design of the microfluidic channel are crafted using Coral Draw software and provided as a drawing or input file to the cutting plotter. A 5 mm-thick PMMA sheet is manually cut into a rectangular shape measuring 40 mm x 50 mm, serving as the baseplate for the microfluidic setup. The baseplate provides good support for the microfluidic channel and helps maintain a rigid flat surface for the channel. The fabrication involves the use of adhesive-coated PVC films in black, white, and transparent colors. To initiate the process, the white PVC film is fed into the cutting plotter, which then precisely cuts the outer boundary according to the PMMA sheet dimensions. The resulting cut white PVC film is affixed to one side (bottom) of the PMMA sheet. Given the transparent nature of PMMA sheets, the white film serves as an optimal background for image capture. Subsequently, the black PVC film is fed into the cutter. The plotter pierces the black film and proceeds to shape the inner contours of the microfluidic device. A large 10 mm x 10 mm well is cut, acting as the inlet. From this larger well, three-way channels are drawn, connecting to three smaller outlet wells, each measuring 5 mm x 5 mm. The channels that link the inlet and outlet wells have a width of 25 mm. The black PVC film, featuring the slotted inlet, channel, and outlet portions, is then attached to the top portion of the PMMA baseplate. The depth of the microchannel is determined by the number of layers of black PVC film stacked on top of each other. In our study, we stacked two layers to achieve a microfluidic channel with an approximate depth of 200 μm.

Finally, a transparent PVC film is fed into the cutting plotter. The plotter cuts the inlet and outlet openings for the device. The transparent adhesive layer is firmly placed on top of the black film, with openings provided for the inlet and outlet, while the rest of the channel portion is completely sealed as shown in Fig. 2. The resulting device was checked for any fluid leakages between the adhesive films and also underwent testing to verify whether the glucose enzyme could traverse from the inlet to the outlet. The enzyme successfully traversed the microfluidic channels through capillary action, reaching the outlet within an average time of 25 s.

### Image capture procedure

Deep learning approaches require training with an initial dataset to achieve optimal classification performance. The effectiveness of the classifier is directly impacted by the dataset’s quality, and improvements can be made by increasing the volume of input data, considering factors such as ambient illumination and camera optics. As a result, a dataset was carefully assembled using a variety of smartphones to recreate and emulate these adverse effects.

**Fig. 3:**
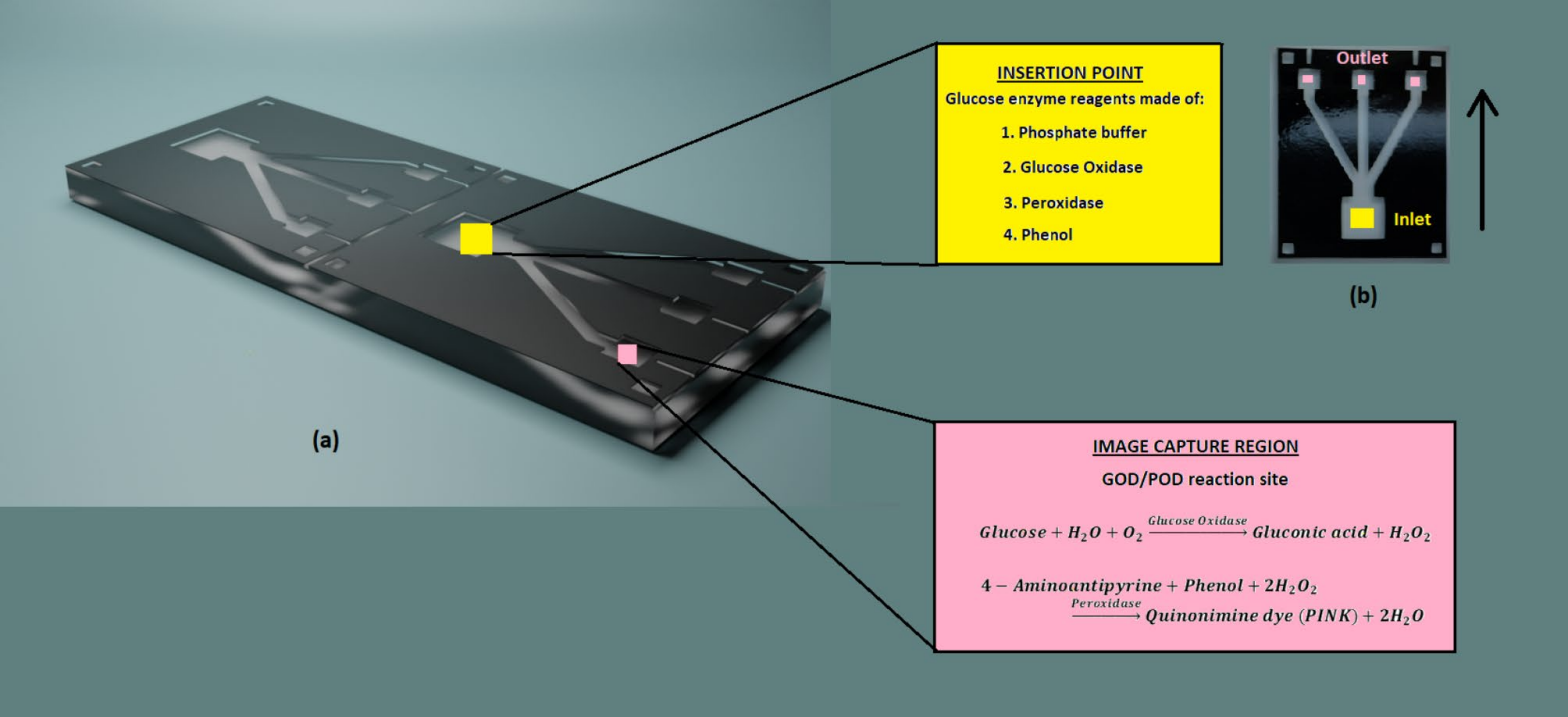
Glucose enzyme insertion point (inlet) and test sample placement point (outlet) in the microfluidic device. (**a**) The insertion points where glucose enzyme containing phosphate buffer, glucose oxidase, peroxide, and Phenol reagents are placed. The image capture region where the glucose test sample is placed. The enzyme traverses along the channel and reaches the outlet well to undergo GOD/POD reaction leading to formation of PINK coloured quinonimine complex. Subsequently, images are captured with the help of smartphone. (**b**) The top view of the fabricated microfluidic device with inlet opening also called the insertion point and output wells (image capture region). The arrow indicates the direction of fluid motion.

Numerous studies have utilized enclosed setups to uphold a fixed distance between the smartphone and the microfluidic device, ensuring a consistent angle of incidence. In our pursuit of maintaining realism, we deliberately omitted artificial illumination and refrained from capturing images within a closed box. Nevertheless, to amass a substantial dataset, we captured images under diverse conditions. The experimental laboratory space benefited from ample natural light through windows and was additionally illuminated by two fluorescent tube lights (Wipro 20 W LED). Three equally spaced locations within the laboratory were designated for placing the microfluidic device to acquire a variety of images. The images were taken under both natural light (fluorescent lights off) and artificial light (fluorescent lights on) using three smartphones from different brands, each with distinct camera optics detailed in Table 1. Focusing on the device outlet region (Fig. 3b), the images were captured in auto mode without any filters, maintaining a consistent distance of approximately 10 cm between the microfluidic device and the smartphone. Given the unique camera setups, optics, and imaging software of each smartphone, the resulting images exhibited considerable diversity. In total, encompassing 16 glucose concentrations, two lighting conditions, three room locations, three smartphone brands, and five images clicked for each instance, we successfully generated a comprehensive dataset comprising 1440 images.

**Table 1:** The optic details of various smartphones used to capture raw images from the microfluidic device.

Brand	Image resolution	Focal length	Optics
iPhone XR	3024 × 4032	4 mm	f/1.8
Moto g62 5G	2296 × 4080	4 mm	f/1.8
Pixel 6a	2268 × 4032	4 mm	f/1.7

### Convolution neural network (CNN)

Deep learning, particularly Convolutional Neural Networks (CNNs), has played a pivotal role in advancing medical image classification and segmentation, thereby ushering in a transformative era for diagnostic accuracy and efficiency. In the realm of medical imaging, where intricate patterns and subtle anomalies are critical for diagnosis, CNNs have excelled in automatically extracting hierarchical features from complex images. Their ability to successfully categorize medical images into distinct classes has found relevance in various applications such as medical image pattern recognition^49^, detection of brain tumour^50^, diagnosis of breast cancer^51^, COVID-19 testing^52^, etc. The sophisticated architecture of CNNs has allowed superior image representation, enabling more accurate and nuanced results compared to traditional methods. Moreover, as medical datasets continued to grow in size and diversity, CNNs exhibited exceptional adaptability, handling vast amounts of data to ensure robust model training.

**Fig. 4:**
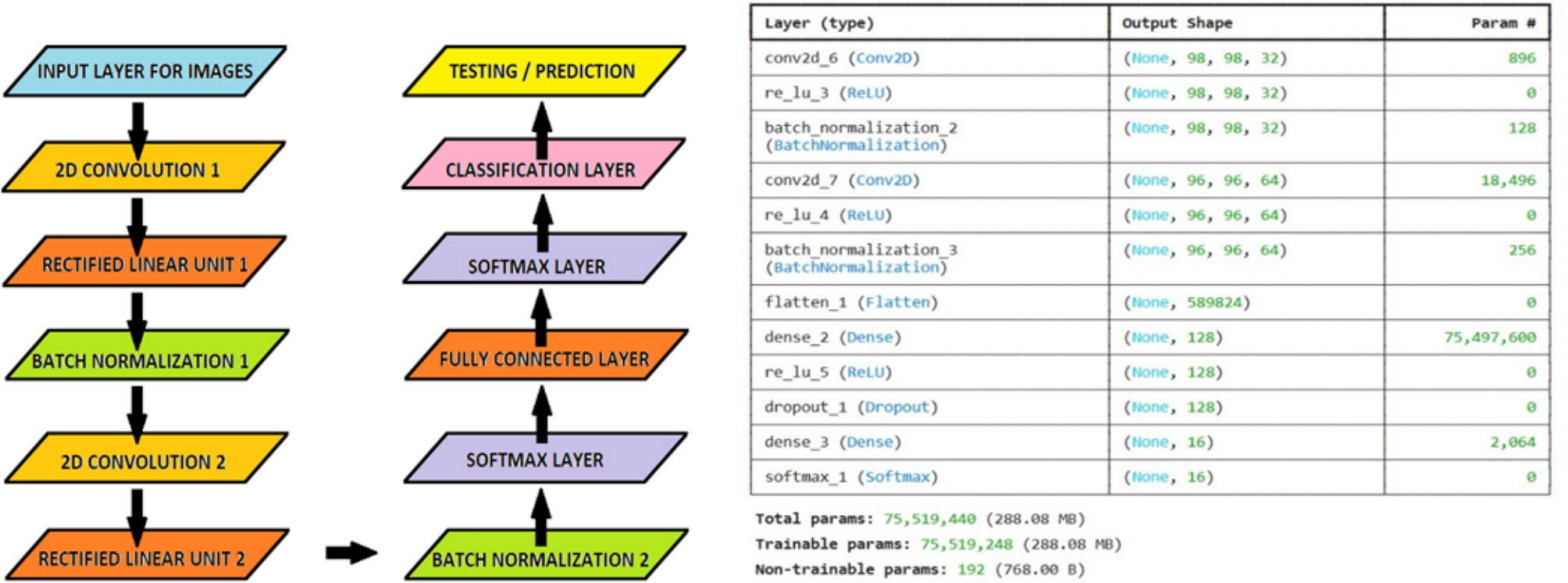
CNN architecture for image classification, showing two convolutional layers with ReLU and batch normalization, followed by fully connected and SoftMax layers. The table provides the output shape and trainable parameters for each layer.

Figure 4 illustrates our CNN model for image classification, which includes two convolutional layers with ReLU activation and batch normalization, followed by fully connected layers, dropout for regularization, and a SoftMax layer for classification. The accompanying data details the output shape and trainable parameters for each layer, highlighting that the model has over 75 million trainable parameters, enabling it to capture image features effectively and achieve high accuracy. The initial layer functions as the input layer, handling the processing of the image dataset. Following this, the 2D convolution layer is implemented, generating a convolution kernel to produce an output tensor. Subsequently, a Rectified Linear Unit layer (ReLU) is applied, featuring an activation function denoted by $\:f\left(x\right)=\text{max}(0,x)$. ReLUs aid the model in accommodating interaction and non-linear effects. A batch normalization layer is then introduced, strategically positioned between layers to expedite the training process and enable a higher learning rate.

The model’s performance is further optimized through the incorporation of successive layers, consisting of 2D convolution, ReLU, and batch normalization layers. These layers are provided to successfully mitigate any overfitting aspect related to classifier prediction. After completing this sequence, a Softmax layer is included, employing a Softmax or normalized exponential function to further normalize the output from the two sets of convolution layers. Semantic segmentation is subsequently conducted through a fully connected layer, followed by the placement of a Softmax layer and a classification layer, facilitating the necessary image predictions. For training and testing purposes, the model utilizes 80% of the entire dataset for training and reserves the remaining 20% for testing.

## Results and discussion

**Fig. 5:**
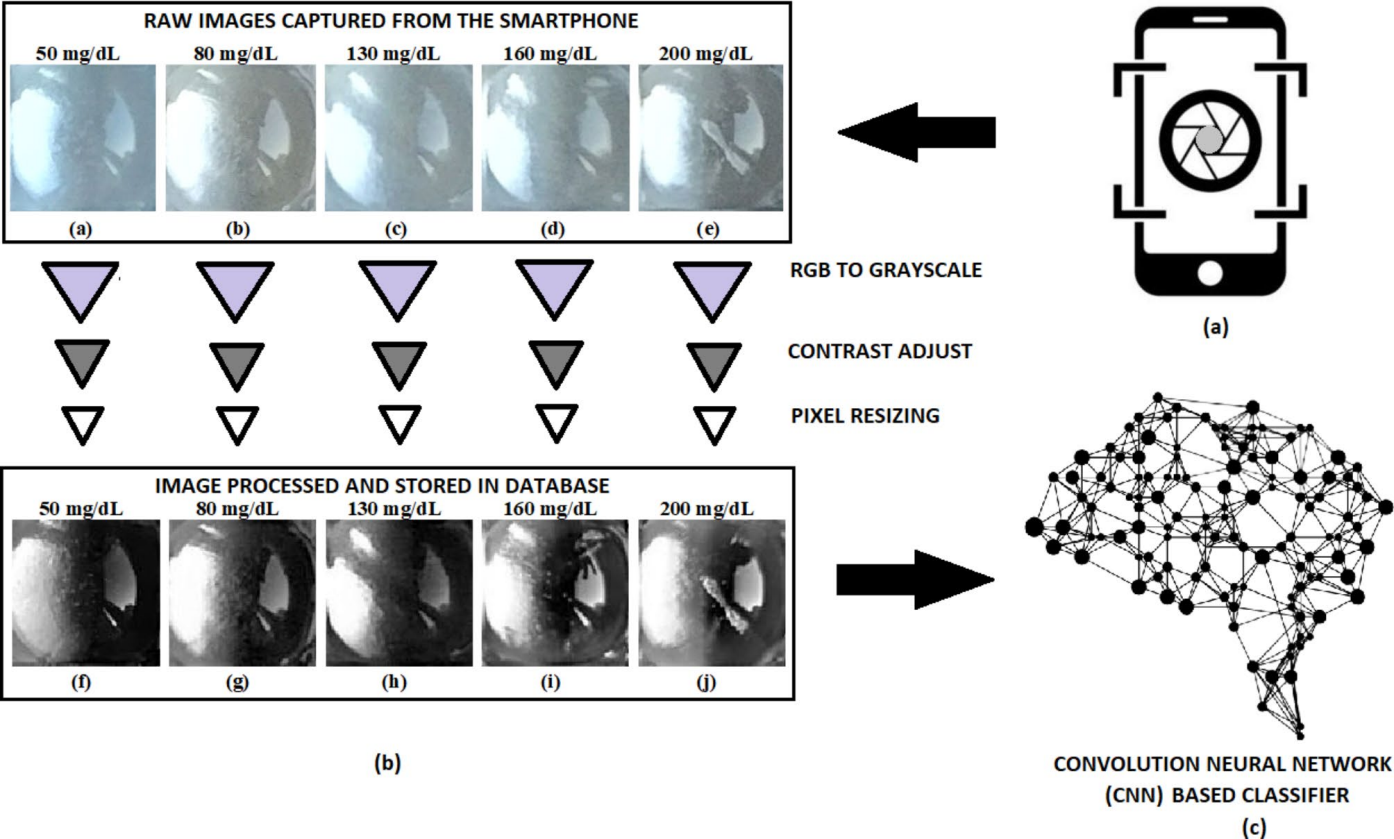
(**a**) Schematic representation of image captured by the smartphone. (**b**) The raw images captured from the smartphone are subjected to three-stage image processing. First, the RBG image is converted to grayscale. Second, the grayscale image is adjusted for contrast. The image is saturated to the bottom 1% and top 1% of pixel values. Third, the saturated images are resized to 100 × 100 pixels to provide consistent input image size for the classifier. (**c**) The processed images are given as input datasets for the Convolution Neural Network (CNN) deep learning classifier and trained. (**c**) Image credit: Bob Holzer.

The detection of glucose in our study relies on the glucose oxidase/peroxidase (GOD-POD) reaction. As shown in Fig. 3a, the inlet well serves as the fixed insertion point, where 57 µL of the glucose enzyme reagent (supplied in the test kit) is introduced. In the outlet well, 1.0 µL of the glucose test solution is positioned. With three outlets available, we can test three glucose samples simultaneously. The glucose enzyme travels through the branched channel and reaches the outlet wells. After the reagent reaches the outlet test wells, the microfluidic chip is lightly shaken (2–3 times) to ensure proper mixing of the test sample and reagent, followed by an incubation period of 10–15 min at room temperature. During the reaction, glucose in the sample undergoes oxidation, resulting in the production of gluconic acid and hydrogen peroxide in the presence of glucose oxidase. The enzyme peroxidase facilitates the oxidative coupling of 4-aminoantipyrine with phenol, generating a pink-colored quinonimine complex. The absorbance of this complex is directly proportional to the concentration of glucose in the sample. Post-reaction, the test samples exhibit consistent color homogeneity and uniformity, distinguishing them from paper-based devices. Following the reaction, images were captured for different concentration values as shown in Fig. 5a.

**Fig. 6:**
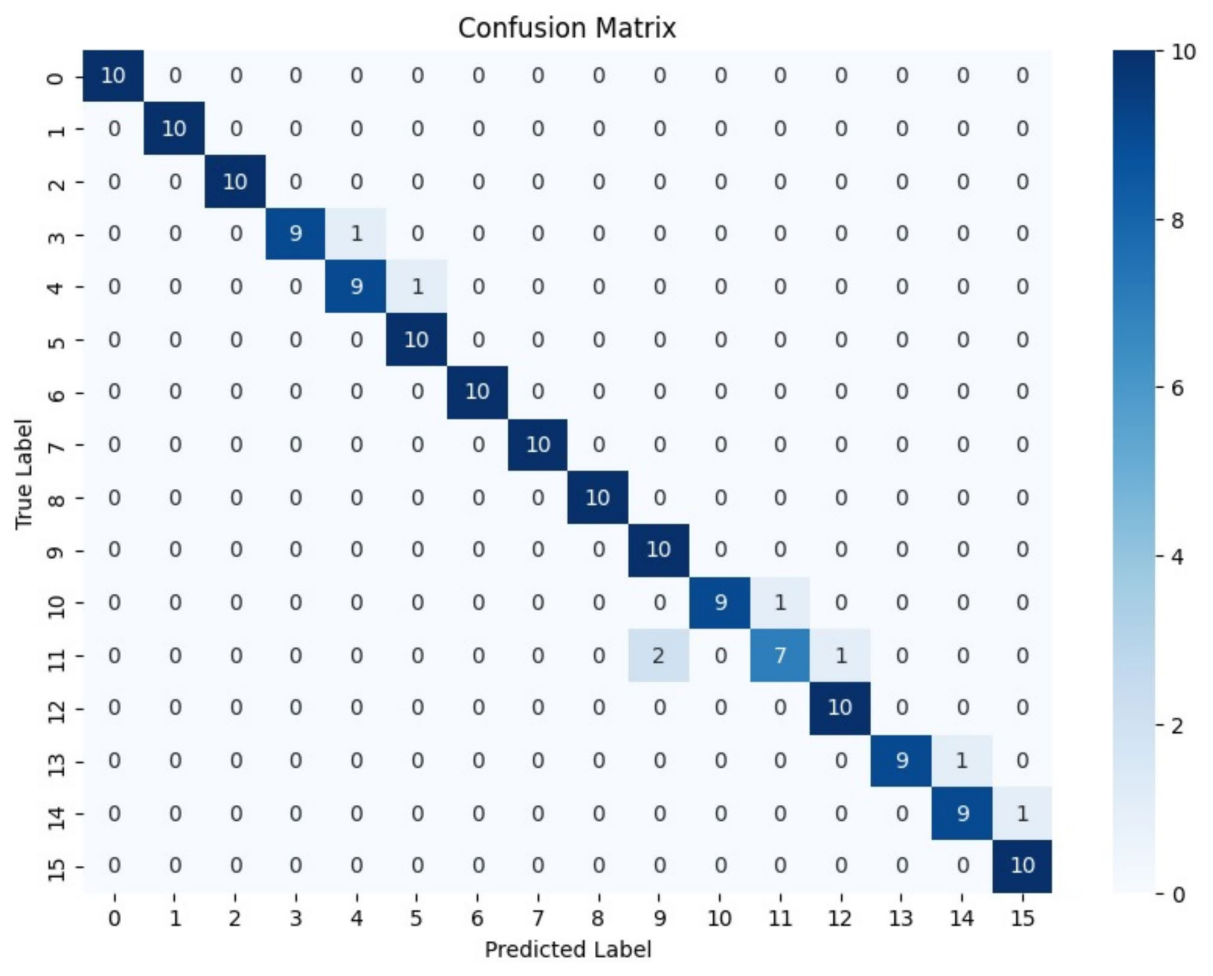
Confusion Matrix from 10-fold cross-validation showing strong classification performance for test set.

To enhance image quality, the RGB images were converted to grayscale. The resulting grayscale images were subjected to contrast adjustment and were ultimately resized to maintain a fixed pixel size as shown in Fig. 5b. These processed images were organized into folders, each labeled with the corresponding glucose concentration values (ranging from 50 mg/dL to 200 mg/dL). Subsequently, these organized images were utilized as input for the deep learning classifier (Fig. 5c). The CNN deep learning classifier was trained as per the input images provided. The confusion matrix shown in Fig. 6 evaluates the model’s performance by comparing true labels (y-axis) to predicted labels (x-axis). The model demonstrates high accuracy, as seen by the strong diagonal, with most classes achieving perfect predictions. However, slight misclassifications are observed in Classes 10 and 11, indicating areas for potential improvement. The use of 10-fold cross-validation ensures the model’s robustness by minimizing overfitting and providing a reliable estimate of its accuracy.

**Fig. 7:**
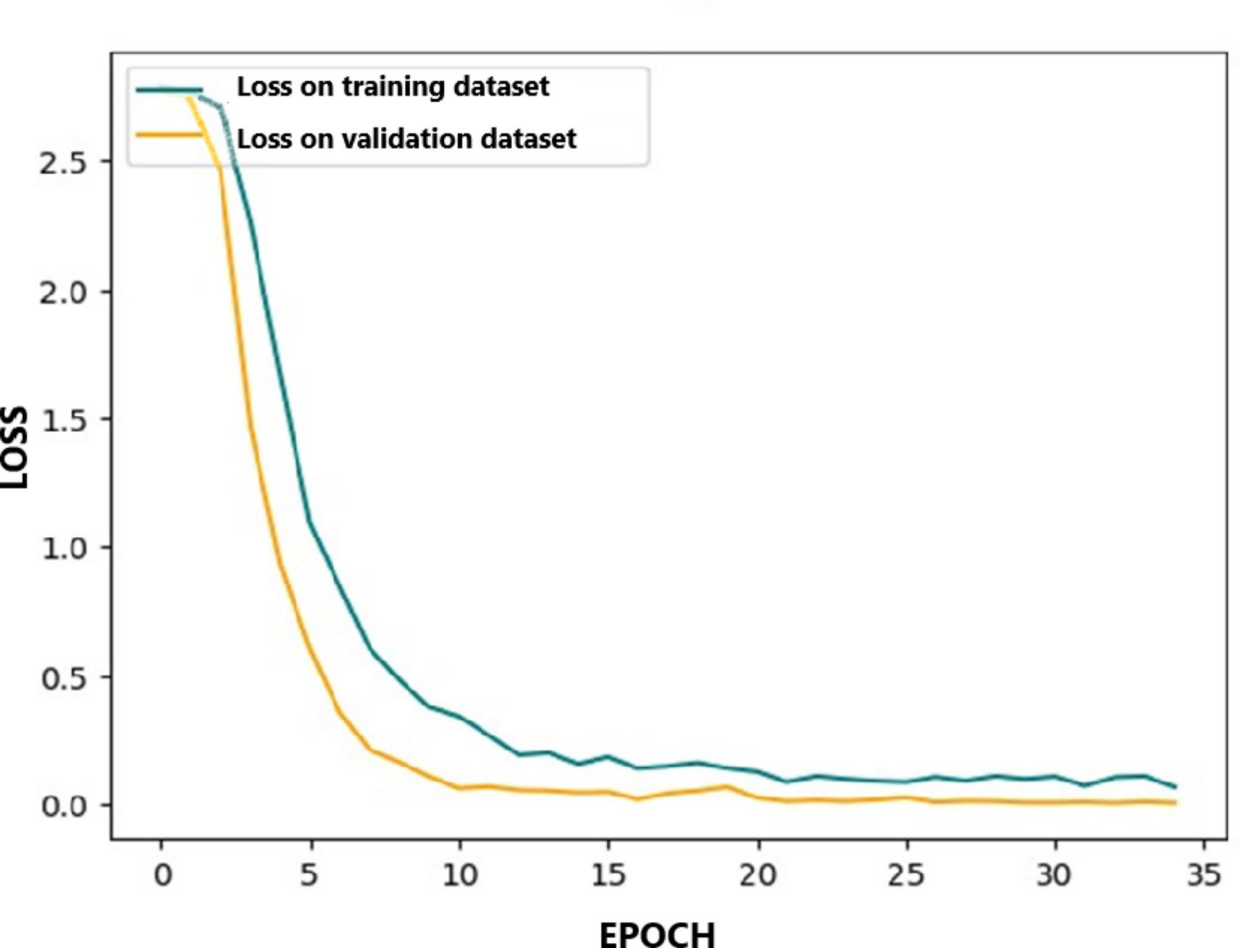
Training (blue) and validation (yellow) loss curves over 35 epochs, showing rapid initial decline and stabilization, with both losses converging near zero, indicating effective model performance.

Figure 7 shows the training and validation loss curves over 35 epochs for the model. The blue curve represents the loss on the training dataset (80% of data), while the yellow curve tracks the loss on the validation dataset (20% of data). Initially, both losses start at a high value and decrease rapidly during the first few epochs, demonstrating significant learning progress. By epoch 5, both curves stabilize, with the training loss continuing to decrease gradually while the validation loss shows a slight improvement. The relatively stable behaviour of the validation loss after epoch 10 suggests that the model is not overfitting, as the validation loss does not increase dramatically. This indicates that the model has generalized well to unseen data and has achieved a satisfactory fit after about 5 epochs, with further improvements in both training and validation loss tapering off after that point. The final convergence around a loss close to zero suggests that the model can effectively predict outcomes on both the training and validation sets.

**Fig. 8:**
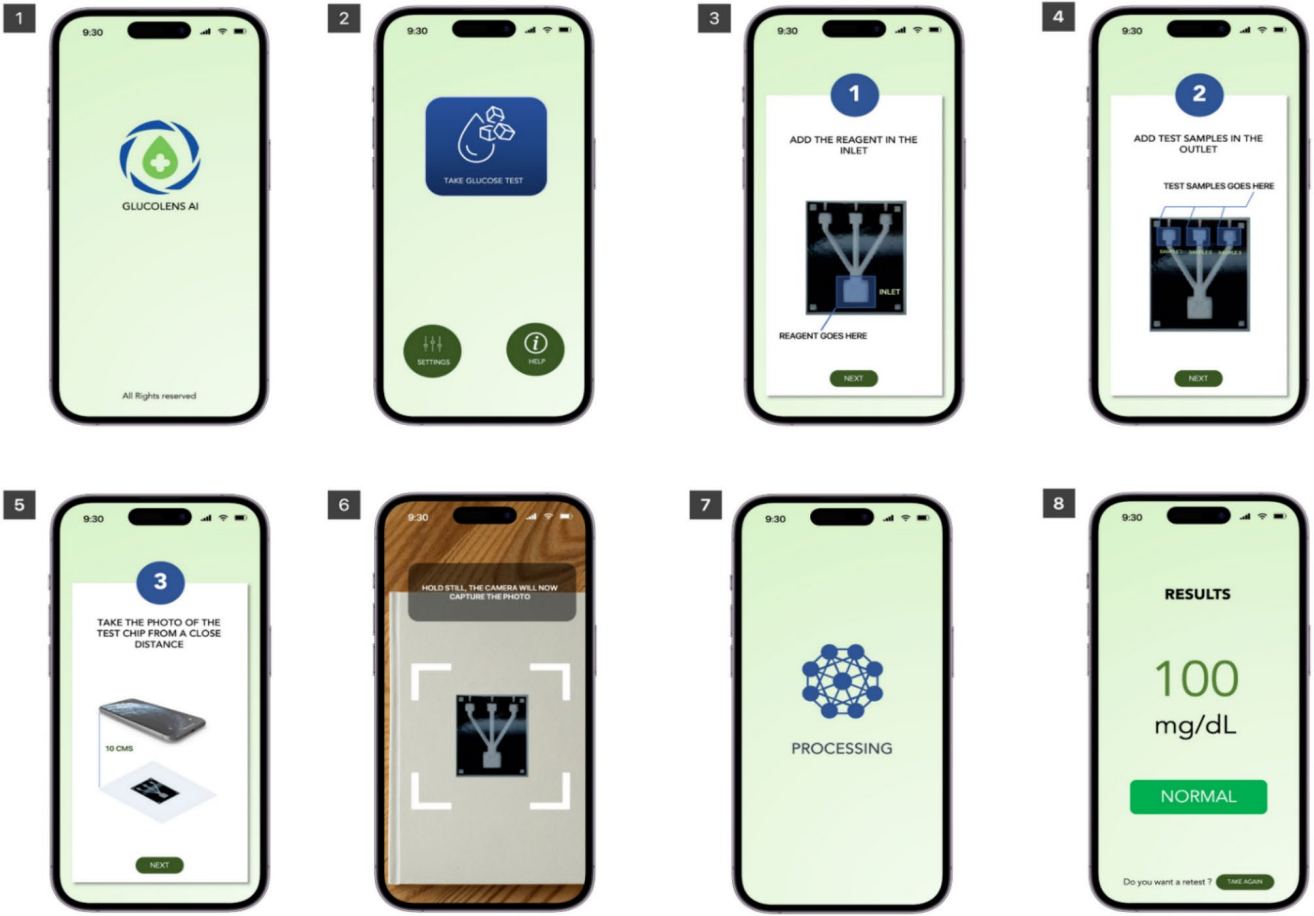
The various stages of digital glucose colorimetric estimation using the “GLUCOLENS AI” smartphone application. In Fig. 1. The application opening stage, 2. The Home page to initiate the glucose test with settings and information buttons, 3. Placing reagent in inlet well, 4. Adding glucose test samples to outlet wells, 5. The application asks the user to place the smartphone camera on top of the device at well-defined distance, 6. The application captures the image of the test device, 7. Processing page and 8. Result display page.

The smartphone application “GLUCOLENS AI” has been developed to employ digital colorimetric techniques for the detection of glucose. Figure 8 elucidates the sequential steps involved in the glucose detection process. Initially, upon opening the application, users are presented with its icon. Upon selecting the glucose detection feature, users are guided through various stages, beginning with the placement of enzymes into the insertion area and the test sample into outlet wells. Furthermore, the application is capable of identifying the proximity of the microfluidic device and prompts users to adjust the distance between the camera and the device accordingly. Once the optimal distance is achieved, the application captures an image. Subsequently, the application identifies the location of the image within the three wells based on markers positioned on the microfluidic device. These images are then processed and transmitted to the CNN classifier for computation. The results are subsequently relayed back to the smartphone via a cloud-based server and an active internet connection. Finally, the application presents the detected glucose concentration levels to the user.

### Classifier validation

To further evaluate the performance of the classifier, four classifier performance parameters were determined. Equations (1)–(4) are used to calculate these parameters.

1 $$\:\text{A}\text{c}\text{c}\text{u}\text{r}\text{a}\text{c}\text{y}=\frac{\text{T}\text{P}+\text{T}\text{N}}{\text{T}\text{P}+\text{T}\text{N}+\text{F}\text{P}+\text{F}\text{N}}$$

2 $$\:\text{R}\text{e}\text{c}\text{a}\text{l}\text{l}=\frac{\text{T}\text{P}}{\text{T}\text{P}+\text{F}\text{N}}$$

3 $$\:\text{P}\text{r}\text{e}\text{c}\text{i}\text{s}\text{i}\text{o}\text{n}=\frac{\text{T}\text{P}}{\text{T}\text{P}+\text{F}\text{P}}$$

4 $$\:\text{F}1\:\text{s}\text{c}\text{o}\text{r}\text{e}=2\times\:\frac{\text{P}\text{r}\text{e}\text{c}\text{i}\text{s}\text{i}\text{o}\text{n}\times\:\text{R}\text{e}\text{c}\text{a}\text{l}\text{l}}{\text{P}\text{r}\text{e}\text{c}\text{i}\text{s}\text{i}\text{o}\text{n}+\text{R}\text{e}\text{c}\text{a}\text{l}\text{l}}$$

Where TP (True Positive) and TN (True Negative) signify the number of accurately predicted positive and negative outcomes, respectively. On the other hand, FP (False Positive) and FN (False Negative) represent the instances of erroneously predicted positive and negative outputs, respectively. Precision, recall, and the F1 score serve as statistical metrics for assessing classifier performance. They involve calculating the ratio of predicted positive and negative outputs. Precision is determined by the ratio of correctly predicted positives to the total positive predictions, while recall is calculated as the ratio of correctly predicted positives to the sum of true positives and false negatives. The F1 score, obtained through the harmonic mean of precision and recall, ranges between 0 and 1, with 1 indicating optimal performance and 0 representing the lowest performance level.

**Fig. 9:**
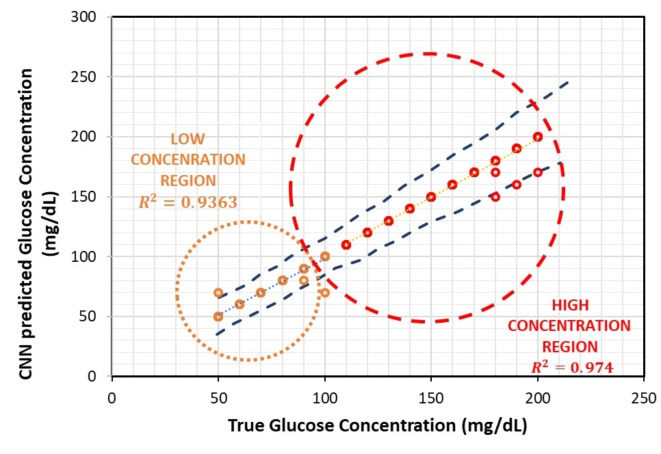
Prediction capability of CNN deep learning classifier for low and high glucose concentration values compared with ISO 15197:2013/2015 gold standard norms given in blue dashed lines. The predictions for low-concentration glucose values produce R^2^ = 0.9363 (Orange dotted circle) and for high concentration the R^2^ = 0.974 (red dotted circle), both within permissible ISO limits.

The classifier was evaluated for various performance parameters and details regarding precision, recall, and F1 score are tabulated in Table 2. The overall accuracy reported for the trained classifier against new images is 95%. Similarly, the overall Precision, Recall, and F1 score are 94%, 93% and 93% respectively. Figure 9 shows the prediction capability of the CNN deep learning classifier for low and high glucose concentration values compared with ISO 15197:2013/2015 gold standard norms. The upper and lower limits are depicted by blue dashed lines. According to ISO standards, it is required that 95% of the results fall within a range of ± 15 mg/dL for glucose concentrations less than 100 mg/dL, and within a range of ± 15% for glucose levels equal to or greater than 100 mg/dL, in comparison to the gold standard result. The actual versus predicted values are plotted and corresponding regression data is obtained for both low and high glucose concentration values. The predictions for low-concentration glucose values produce R^2^ = 0.9363 (Orange dotted circle) and for high concentration the R^2^ = 0.974 (red dotted circle), both within permissible ISO limits. This collectively signifies the classifier’s overall exceptional prediction capability.

**Table 2:** Performance evaluation of CNN classifier based on Precision, recall, and F1 score.

Sample	Precision	Recall	F1 -score
50	1	0.93	0.97
60	1	1	1
70	1	1	1
80	1	1	1
90	1	0.85	0.91
100	1	0.85	0.91
110	1	1	1
120	1	1	1
130	1	1	1
140	1	0.92	0.96
150	1	0.85	0.91
160	1	1	1
170	1	1	1
180	1	0.7	0.83
190	1	0.85	0.91
200	1	0.92	0.96

The primary limitation of the current study lies in its failure to account for the impact of hematocrit content on the sampled specimens during glucose estimation. There is also a need to incorporate a membrane filtration system that effectively separates hematocrit content and plasma from blood samples if hematocrit contents are to be considered. The methodology employed in this investigation mainly involves the initial creation of a dataset comprising samples with predetermined glucose concentrations. Subsequently, these samples are utilized to train deep learning models, leveraging the feature engineering capabilities of deep neural networks to extract pertinent features from images that will be obtained from actual patient test samples.

Finally, our study involves a comparison and summarization of our results with recent literature pertinent to glucose estimation, as illustrated in Table 3. We examined reduction, non-enzymatic, and enzymatic-based glucose detection strategies for this purpose. Table 3 reveals that the majority of studies have gathered images in a controlled environment by placing the test device within a well-regulated illumination box or setting. Additionally, many studies utilized an additional instrument to support the smartphone and test device. In contrast, our study does not capture images under controlled illumination settings and is truly instrument-free in its usage. Regarding image analysis, most studies rely on manual feature extraction from the image properties, whereas our study utilizes CNN, which automatically extracts features from the input data provided. The performance of CNN is deemed reasonable compared to recent classifiers such as random forest regression (RFR), linear discriminate analysis (LDA), multi-linear regression (MLR), and ensemble bagging classifier (EBC). It’s noteworthy that with continued image capture and data collection, the accuracy of this pre-trained CNN classifier is anticipated to experience significant improvement over time.

**Table 3:** Comparison of glucose estimation studies carried out by previous researchers and present studies for different conditions.

Analyte	Principle	Detection range (mg/dL)	Multiple smartphones utilized	Illumination controlled*	Instrument^#^	Feature extraction	Machine learning model	Best performance (%)	References
D + Glucose	Polyaniline nanoparticle reduction	0–1080	NO	YES	FIXED	MANUAL	RFR	92.2	^41^
D + Glucose	Gold nanoparticle, Non-enzymatic	9–162	NO	YES	FIXED	MANUAL	LDA	93.63	^42^
Glucose-Artificial saliva	GOX-HRP enzyme reaction with TMB/KI/KI + Chi	0–180	YES	YES	FIXED	MANUAL	LDA	98.24 (TMB)	^40^
Blood plasma	GOX-HRP/KI	50–400	YES	YES	FIXED	MANUAL	MLR	98.11	^39^
Glucose solution	GOX-HRP/C_11_H_13_N_3_O, C_7_H_6_O_3_	10–400	NO	YES	FIXED	MANUAL	MLR	96.96	^43^
Plasma glucose	GOX-HRP with TMB/KI	0–540	YES	NO	FREE	MANUAL	EBC	95.41 (TMB)	^53^
Glucose solution	GOX-HRP/C_11_H_13_N_3_O, C_7_H_6_O	50–200	YES	NO	FREE	AUTOMATIC	CNN	95.00	Present study

*Illumination is provided inside an enclosed box/setting.

#The smartphone and test device are either attached to an illumination box (FIXED) or not attached (FREE).

### Cost estimation

In addition to evaluating the scientific effectiveness, we also determined the cost per test by carefully considering both business-related factors and technical aspects, consulting experts in the field for insights. The breakdown of costs per test was projected by analyzing the individual components. Notably, the only consumable required for the test is a ready-to-use adhesive-coated poly-vinyl film stacked on a PMMA sheet microfluidic device. This consumable must be used for each test and then disposed of following appropriate biosafety protocols. It is assumed that individual users or community-based users will have access to at least one pre-owned smartphone with a built-in camera. Factoring in various cost elements inherent to the business supply chain, the estimated cost per test is approximately 0.1 USD.

## Conclusion

In this study, a novel microfluidic device was constructed by layering adhesive poly-vinyl films having micro-channel contours precision cut using a cutter printer and placed on a poly methyl methacrylate (PMMA) base-sheet. In our investigation, glucose detection hinges on the glucose oxidase/peroxidase (GOD-POD) reaction. The inlet well functions as the designated entry point, where 57 µL of the glucose enzyme reagent (provided in the test kit) is introduced. The reagent is divided into 3 parts (19 µL each). Positioned in the outlet well is a volume of 1 µL of the glucose test solution. With three outlets at disposal, simultaneous testing of three glucose samples becomes feasible. Capillary action propels the enzyme, aiding its interaction with the glucose sample through the GOD/POD reaction, replicating the conditions of the gold standard laboratory test, and generating a pink-colored quinonimine complex. The absorbance of this complex is directly proportional to the concentration of glucose in the sample. Three evenly distributed positions within the laboratory were assigned for the placement of the microfluidic device to capture a diverse range of images. The photographs were captured under both natural light (with fluorescent lights turned off) and artificial light (with fluorescent lights turned on) using three smartphones from different brands, each equipped with unique camera optics. The processed images were systematically arranged into folders, with each labeled according to the corresponding glucose concentration values, spanning from 50 mg/dL to 200 mg/dL. These meticulously organized images were then employed as input for the CNN deep learning classifier. The CNN deep learning classifier underwent training based on the provided input images. The validation of the trained network was carried out with new images and performance tests against various parameters. The trained classifier exhibited an overall accuracy of 95% when tested against new images. Likewise, the overall Precision, Recall, and F1 scores achieved were 94%, 93%, and 93%, respectively. The correlation between the true labels and predicted labels was determined by comparing with ISO standard norms. The predictions for low-concentration glucose values produce R^2^ = 0.9363 and that for high concentration is R^2^ = 0.974. Collectively, this indicated the overall exceptional prediction capability of the classifier. To make the entire glucose colorimetric detection user-friendly and take into consideration the POC aspect, a smartphone application called “GLUCOLENS AI” was developed that performed various tasks like providing users instruction, image capture, image processing, and communication with CNN classifier embedded in cloud-based platform. It is important to note that with the continued capture of images and collection of data, the accuracy of this pre-trained classifier is expected to witness a substantial improvement over time.

With this, we conclude that our microfluidic device characterized by affordability, portability, and user-friendly design, stands out as an accessible and convenient solution for POC applications. The system required no specialized training and functioned seamlessly without the need for skilled professionals. Employing a self-contained colorimetric detection method based on a smartphone camera, the device is genuinely instrument-free and eliminates dependencies on additional components like casings, plastic boxes, or mobile holding platforms. With the incorporation of deep-learning models trained on current and future datasets, accurate gold-standard colorimetric detection on smartphones is possible with minimal computational demand, ensuring glucose estimation independently of external calibration curves developed in controlled settings. Finally, this system can consistently deliver accurate and reliable glucose estimations within established standards as proved in our study.

## Data Availability

Data sets generated during the current study are available from the corresponding author upon reasonable request.
